# Models of upland species’ distributions are improved by accounting for geodiversity

**DOI:** 10.1007/s10980-018-0723-z

**Published:** 2018-10-28

**Authors:** Joseph J. Bailey, Doreen S. Boyd, Richard Field

**Affiliations:** 1 School of Geography, University of Nottingham, University Park, Nottingham, NG7 2RD UK; 2 School of Humanities, Religion and Philosophy, York St John University, Lord Mayor’s Walk, York, YO31 7EX UK

**Keywords:** Biodiversity, Conserving Nature’s Stage, Geodiversity, Geomorphometry, Heterogeneity, Landscape, Scotland, Species distribution modelling

## Abstract

**Context:**

Recent research suggests that novel geodiversity data on landforms, hydrology and surface materials can improve biodiversity models at the landscape scale by quantifying abiotic variability more effectively than commonly used measures of spatial heterogeneity. However, few studies consider whether these variables can account for, and improve our understanding of, species’ distributions.

**Objectives:**

Assess the role of geodiversity components as macro-scale controls of plant species’ distributions in a montane landscape.

**Methods:**

We used an innovative approach to quantifying a landscape, creating an ecologically meaningful geodiversity dataset that accounted for hydrology, morphometry (landforms derived from geomorphometric techniques), and soil parent material (data from expert sources). We compared models with geodiversity to those just using topographic metrics (e.g. slope and elevation) and climate data. Species distribution models (SDMs) were produced for ‘rare’ (N = 76) and ‘common’ (N = 505) plant species at 1 km^2^ resolution for the Cairngorms National Park, Scotland.

**Results:**

The addition of automatically produced landform geodiversity data and hydrological features to a basic SDM (climate, elevation, and slope) resulted in a significant improvement in model fit across all common species’ distribution models. Adding further geodiversity data on surface materials resulted in a less consistent statistical improvement, but added considerable conceptual value to many individual rare and common SDMs.

**Conclusions:**

The geodiversity data used here helped us capture the abiotic environment’s heterogeneity and allowed for explicit links between the geophysical landscape and species’ ecology. It is encouraging that relatively simple and easily produced geodiversity data have the potential to improve SDMs. Our findings have important implications for applied conservation and support the need to consider geodiversity in management.

**Electronic supplementary material:**

The online version of this article (10.1007/s10980-018-0723-z) contains supplementary material, which is available to authorized users.

## Introduction

An intimate relationship exists between living things and the geophysical land surface (Lawler et al. 2015), which appears to be more pronounced at the landscape scale than at larger geographic extents (Hjort et al. 2012; Stein et al. 2014; Bailey et al. 2017; Tukiainen et al. 2017a). Capturing this geophysical diversity, or ‘geodiversity’, is important for biodiversity conservation because geodiverse areas may facilitate species’ persistence and adaptation to climate change (Anderson and Ferree 2010; Albano 2015; Ordonez et al. 2016; Magness et al. 2018; Suggitt et al. 2018). Developing more ecologically meaningful ways to quantify geodiversity is therefore essential to help inform conservation planning and adaptation strategies for the future (e.g. Hagerman et al. 2010; Anderson et al. 2014; Theobald et al. 2015).

Macroecological work has tended to be conducted at larger extents than that of the landscape, and either largely focus on species richness, which itself tells us mainly about common species (Gaston 2010), or on species distribution modelling (SDM) using climatic envelopes. Such SDMs suffer from the unrealistic assumption that species’ realized niches are the same as their fundamental niches (Kearney et al. 2010) and, despite recent efforts to include relatively crude geophysical data at broad scales (Title and Bemmels 2017), studies using such data in SDMs at landscape scales are limited. Meanwhile, ecological studies using small plots across a limited extent can be too autecological, missing landscape-scale drivers of observed biodiversity and species’ distributions (Boyd et al. 2013). To bridge this gap, we need predictors that are capable of capturing ecologically relevant geophysical characteristics of the landscape.

This gap has often been addressed using measures of spatial environmental heterogeneity, which describe the diversity of the physical environment in a very coarse way. The relationship between environmental heterogeneity and both biodiversity and species’ distributions is well documented, especially at the landscape scale where climate tends to be less variable through space than it would be across larger areas (Stein et al. 2014). Heterogeneity metrics are varied, but most commonly include coarse topographic measures such as openness, and mean and range of elevation and slope. Although their value in macroecology has been shown repeatedly across taxa and scales (Pausas et al. 2003; Dufour et al. 2006; Jeremy and Lundholm 2009; Parks and Mulligan 2010; Stein 2015), these relatively crude measures may oversimplify the physical environment, thus precluding a more advanced ecological understanding of relationships that have been known for some time (Hjort et al. 2012; Lawler et al. 2015).

‘Geodiversity’ may be defined as the natural range of hydrological, geomorphological, and geological features, comprising surface and sub-surface materials and landforms (Hjort et al. 2012; Gray 2013). The body of research on geodiversity–biodiversity relationships has expanded in recent years (Gray 2013; Lawler et al. 2015). These works share a common goal to more effectively link the living and non-living constituents of the landscape and, in doing so, adhere closely to original definitions of the ‘ecosystem’ (Tansley 1935; Willis 1997). In capturing geodiversity, we should be able to produce a more nuanced view of the landscape and further our understanding of, and ability to manage, biodiversity. In Finland, diversity metrics calculated from expertly mapped geodiversity data (Hjort et al. 2012) have demonstrated biodiversity–geodiversity links at the landscape scale. Meanwhile, similar patterns have been reported at multiple scales across Great Britain (e.g. grain sizes of 1 km^2^ and 100 km^2^ and several extents of circular areas with diameters between 25 km and 250 km), where geomorphometric methods (automated landform mapping using digital elevation models) were used to quantify landform coverage in relation to biodiversity (Bailey et al. 2017).

Geophysical features, or ‘geofeatures’ (e.g. geological types, landforms and hydrological features), relating to both landform morphology (i.e. the geometry of the landscape) and surface materials, are directly relevant to species’ distributions and biodiversity through their implicit links to abiotic processes (e.g. disturbance, weathering, fine-scale hydrology), properties (e.g. nutrient levels), and settings (e.g. microclimate, connectivity from rivers). These links are fundamental to ecological theories surrounding the niche (Peterson et al. 2011), including biotic heterogeneity (Tuanmu and Jetz 2015), and local resource availability (Dufour et al. 2006; Viles et al. 2008; Bétard 2013; le Roux et al. 2013; Hjort et al. 2015).

Automatically mapping landforms across large extents for biodiversity modelling is now relatively straightforward using geomorphometric techniques (e.g. Bailey et al. 2017). However, using only shape overlooks the importance of surface materials, which implicitly capture important ecology-relevant information because of the genesis (e.g. glacial; fluvial) of a landform. Expertly mapped, explicit geomorphological features capture this in a way that DEM-based landform mapping does not, but expert geomorphological maps (e.g. in Hjort and Luoto 2010) are time-consuming to produce and exist in very few places worldwide. Combining automated landform maps with existing, widely available surface material maps should produce more ecologically meaningful data than either in isolation. This technique is extendable across large areas, without the need for extensive field mapping. However, such semi-automated mapping has not yet been done in biodiversity science, though some research in geomorphology points to the possibilities (Anders et al. 2009; Seijmonsbergen et al. 2014).

We therefore aim to test the ability of geodiversity variables to improve models of individual plant species’ distributions across a sensitive upland landscape—the Cairngorms National Park, Scotland, UK. As part of a macroecological approach, we first consider a traditional SDM built on climate and topography data, and then add various geodiversity data, including the combined landform-parent material data to account for source and mineralogy. We determine the explanatory power of the geodiversity variables over and above commonly used, coarse measures of environmental heterogeneity. The Cairngorms provide a very suitable place in which to examine these techniques and relationships at the landscape scale, especially given the availability of a recently compiled database of rare species (broadly defined). This allows us to develop an improved understanding of rare and common species’ relationships with geodiversity at the landscape scale.

## Methods

### Study area

**Fig. 1:**
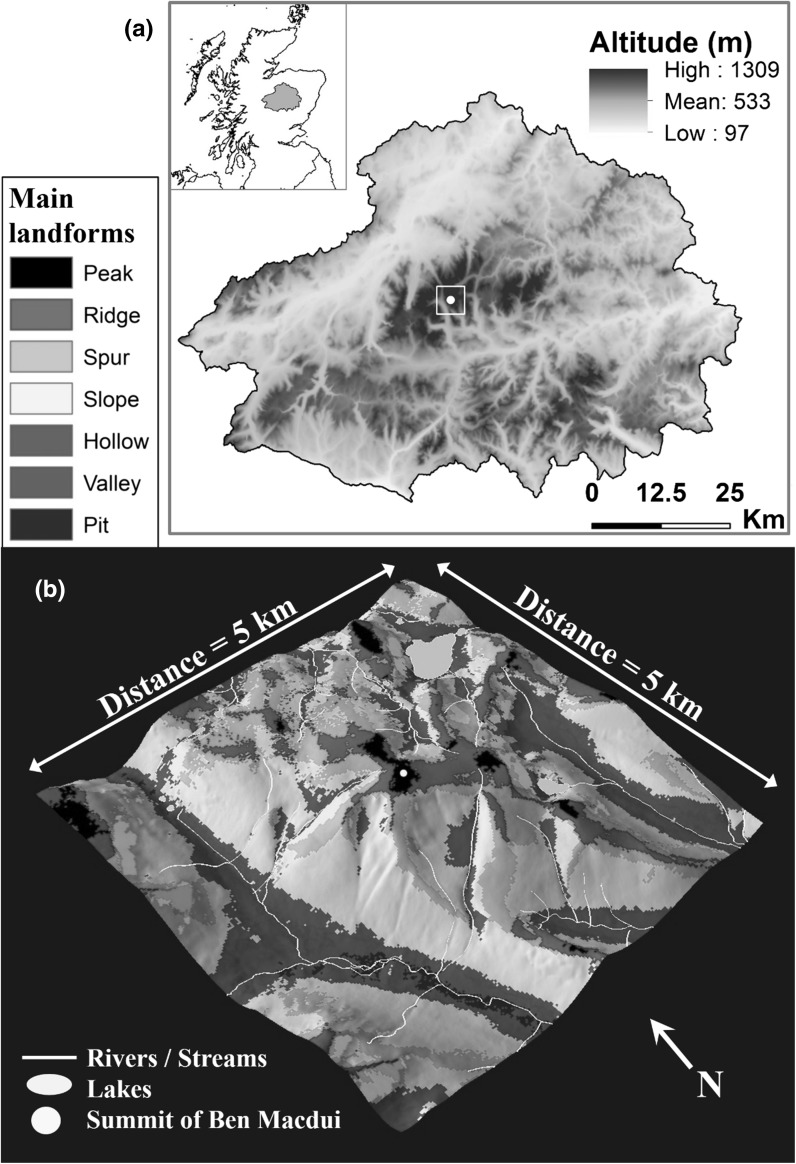
a Elevation map of the Cairngorms National Park (CNP) with an inset showing CNP shaded grey within Scotland; **b** A 3D visualisation of the geomorphometric landform classification (produced using r.geomorphon in GRASS GIS 7.1) produced in ArcScene 10.3: these data were aggregated to the 1 km^2^ grid used in this study (see Appendix S2b for aggregated map examples). **b** is centred over Ben Macdui (altitude = 1309 m—the highest point in the Cairngorms), which is shown with the yellow circle in the centre of the image. Rivers and lakes are shown as white lines and polygons, respectively. The map in **a** uses a 10 m elevation raster, derived from Intermap Technologies NEXTMap (accessed via NERC Earth Observation Data Centre; Table 1), which was aggregated to the 1 km^2^ resolution for analyses

The Cairngorms National Park, Scotland, is the largest (area = 4528 km^2^) and highest national park in Great Britain (Fig. 1a; also see Appendix S1 in Supporting Information). It is valuable for nature conservation (Nethersole-Thompson et al. 1974; Gimingham 2002; Shaw and Thompson 2006) and about half of its area is designated as internationally important under European law (Amphlett 2012). Both the sensitivity of this landscape and the value of its geoheritage have been recognised for some time (Gordon et al. 1998, 2001; Haynes et al. 1998; Gordon and Wignall 2006; Kirkbride and Gordon 2010). The central mountains form a number of granite plateaus, with deep passes in between, whilst in the wider national park, Dalradian and Devonian sedimentary rocks define the underlying geology (Gordon and Wignall 2006). Soils are derived from underlying solid geology and superficial deposits (Bruneau 2006). This results in a complex abiotic environment, exhibiting a substantial range in elevation (mean elevation of 533 m; min = 97 m; max = 1309 m) and slope (mean = 11°; min = 0°; max = 72°) (statistics derived from a 10 × 10 m DEM).

Landforms that largely pre-date the last glaciation (Late Devensian/Weichselian) are extensive and include palaeosurfaces and breaks of slope, topographic basins, shallow plateau valleys, domes and tors (Hall et al. 2013). Glacial landforms are marked by sudden breaks from the gentler pre-glacial mountain forms, and include corries, glacial troughs and glacially breached watersheds (Sugden 1968; Gordon and Sutherland 1993). Periglacial features such as solifluction lobes and boulder fields occur on upper slopes, and moraines and glaciofluvial deposits on lower ground in the valleys and straths (large valleys) (Kirkbride and Gordon 2010). These create a geodiverse landscape.

Windy, cool, humid conditions dominate the Cairngorms, with higher areas experiencing weather and climate similar to those of the alpine semi-tundra (McClatchey 1996; Gordon et al. 1998; Gimingham 2002). Mean precipitation ranges from 800 mm to 1500 mm per year and is broadly linked to altitude. However, the eastern areas are typically drier because of the predominant westerly direction of approach of Scotland’s weather, meaning that much precipitation falls when weather systems encounter the western mountains. Alpine, low alpine, and sub-alpine habitats exist at higher elevations. More widely, heathland (heather moorland) and native pinewoods dominate the area. Snow-bed communities are relatively common and include rare and specialized liverworts and mosses, for example, especially on the high plateaus.

### Data

All predictor sets and subsets used in the modelling are summarised in Table 1. Except for geomorphometric analyses (see below), all data for the Cairngorms National Park study area were processed and joined to 1 km^2^ British National Grid (BNG) cells (n = 4774) using ArcGIS 10.3 and subsequently processed and analysed in R (R Core Team 2018). Grid cells with < 75% land area in the national park boundary or those that had no species occurrence data were removed.

**Table 1:** Details of the variables within each predictor set

Predictor class	[Category] Variables	Original resolution or map scale	Value per 1 × 1 km grid cell	Source
Climate	Bio1 (annual mean temperature) and Bio12 (annual precipitation)	30 arc seconds	Mean	CHELSA
Topography	Elevation	10 m	Mean and SD	NEXTMap data (Intermap via NEODC)
	Slope	10 m	Mean and SD	NEXTMap data (Intermap via NEODC)
Geodiversity components (GDCs)	[Landforms] Ridges, slopes, spurs, peaks, pits, hollows, valleys, and flat areas	10 m	Areal coverage	Derived from NEXTMap data (Intermap via NEODC) in GRASS GIS 7.1^a^
	[Hydrology] River length	1:50,000	Total length	OS Strategi via Edina Digimap
	[Hydrology] Lake area	1:50,000	Areal coverage	OS Strategi via Edina Digimap
	[Materials] Parent material source	1:50,000	Areal coverage	British Geological Survey (BGS) under Academic License
	[Materials] Mineralogy	1:50,000	Areal coverage	British Geological Survey (BGS) under Academic License
	*[Combined] Parent material source *×* landforms*	*See above*	*Areal coverage*	*See above*
	*[Combined] Mineralogy *×* landforms*	*See above*	*Areal coverage*	*See above*

Italicised content = data produced by combining other predictors. The predictors used in each model are detailed in Table 2

*OS* Ordnance Survey, *SD* standard deviation

CHELSA (http://chelsa-climate.org/bioclim/) (Karger et al. 2017); Intermap (www.intermap.com); NEODC = National Environment Research Council (United Kingdom) Earth Observation Data Centre (www.neodc.nerc.ac.uk)

^a^Jasiewicz and Stepinski (2013)

#### Climate and topography data

The mean and standard deviation in elevation were calculated for each grid cell from the NEXTMap 5 × 5 m digital elevation model, which we resampled to 10 × 10 m to reduce noise (Table 1). The mean of CHELSA’s (Karger et al. 2017) bioclimatic variables 1 (bio1; annual mean temperature) and 12 (bio12; annual precipitation), with an original resolution of 30 arc-seconds, were calculated for each 1 km^2^ grid cell.

#### Geodiversity data

We compiled geodiversity data (landforms, parent material, and hydrology) from existing datasets. River and lakes data were obtained under open license from the Ordnance Survey’s ‘OpenData’ service (Appendix S2a). We checked these data against 1:50,000 OS base maps and added smaller rivers where appropriate (by digitisation in ArcMap). Total river length and lake area were calculated for each 1 km^2^ grid cell.

Using the 1:50,000 British Geological Survey (BGS) Soil-Parent Material Database, under academic license, two key datasets were extracted: source (relates to material genesis and rock type, e.g. sedimentary alluvial, sedimentary glaciolacustrine, igneous intrusive; number of classes = 28) and mineralogy (e.g. basic, acid, calcium carbonate; number of classes = 18).

The geology of the whole area has been systematically mapped by the British Geological Survey, but since geomorphological mapping is only available for the core mountain area (Kirkbride and Gordon 2010), a geomorphometric approach was adopted to categorise landforms across the whole of the national park. We produced morphological landform coverage data using geomorphometry. Specifically, we used the r.geomorphon algorithm developed by Jasiewicz and Stepinski (2013), in GRASS GIS 7.1 (GRASS Development Team 2018), which uses relational geometry to define a grid cell as belonging to a particular landform class. Jasiewicz and Stepinski’s landform definitions (also see their Fig. 3, p. 150) were maintained in our study and the following landform features were mapped (also see our Fig. 1b and Appendix S2b): *peak* (slope declining away from focal grid cell in all directions), *ridge* (slope declining on either side), *shoulder* (a declining slope leading from an area of flat ground), *spur* (a ridge oriented in a downward direction from the top of a slope), *slope* (consistently inclining or declining slope), *footslope* (a declining slope leading into an area of flat ground), *hollow* (a depressed area within a slope), *valley* (slope inclining on either side), *flat area* (consistent absence of slope within focal area), and *pit* (slope declining towards from focal grid cell from all directions).

Raster grid cells in the geomorphometry data were removed if they overlapped with known lakes. This was particularly common for ‘pits’, which represent depressions in the landscape that are likely to be hydrologically significant (perhaps kettle holes, bogs, or ponds, for example), but are not mapped as hydrological features.

The surface parent material data on source and mineralogy were each combined with these landform (morphology) data using GIS, so that material and landform were explicitly accounted for by novel variables. This produced two datasets accounting for the coverage of *landform*–*material* combinations, which were used as predictor sets in separate species’ distribution models. Examples of these combined landform–material variables for the *landform*-*source* dataset were: coverage of glaciofluvial ridge, alluvial terrace slope, glacigenic valley, and igneous spur for the genesis dataset (n = 107 such combinations exist within the landform–source dataset). For the *landform*–*mineralogy* dataset, for e.g.: clay silica valley; calcium carbonate hollow; basic slope (137 such combinations exist within the landform–mineralogy dataset). These data are detailed in Appendix S3.

#### Species data

Species’ occurrence data were provided at a resolution of 1 km^2^ (British National Grid cells) by the Botanical Society of Britain and Ireland (BSBI), via the Distribution Database. The BSBI hosts a single database (the ‘Distribution Database’) to which data are contributed by its volunteers and coordinators—who are strongly encouraged to use unbiased sampling (Groom et al. 2011). We used accepted data records (those verified within the database) from the last 20 years and rejected any species occurring in fewer than ten grid cells. The Cairngorms ‘Rare Plant Register’ (RPR) was used to identify rare species records for the area (Amphlett 2012), whilst other species were classified as ‘common’ in this study.

The definition of ‘rare’ species in the RPR is broad and comprises species that are listed in the UK Red List, UK Biodiversity Action Plan, Scottish Biodiversity List and Wildlife and Countryside Act, or species that are considered to be an endemic, native or archaeophyte within the Cairngorms, nationally rare or scarce; or a European Protected species (Amphlett 2012). For archaeophytes, only those that are rare in the Cairngorms or of cultural significance are included. The species in this list have no consistent ecological difference compared to the ‘common’ species, but their inclusion provides conceptual knowledge around species considered rare in this landscape.

**Fig. 2:**
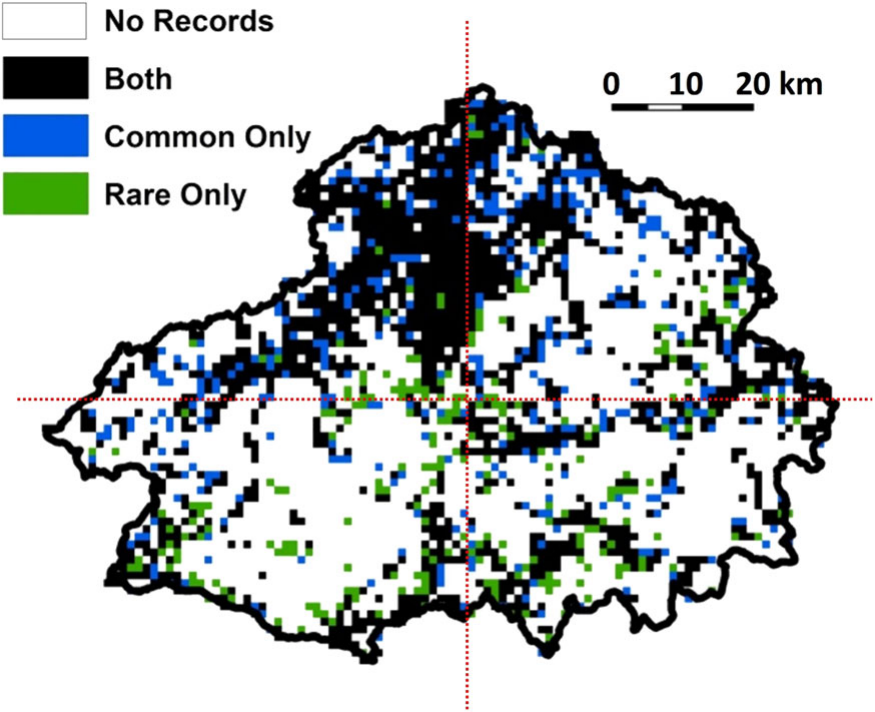
The distribution of rare and common species occurrences on the 1 × 1 km grid used (blue = common records only; green = rare only; black = both) within the Cairngorms National Park. (Colour figure online)

Data quantities were sufficient to run models for 76 ‘rare’ species (covering 1640 grid cells; 34.6%) and 505 ‘common’ species (1757 grid cells; 36.8%). Many cells contained only rare species, which can be explained by the broad definition of rare species used by this dataset, within which some species will be locally common. Additionally, the BSBI may carry out surveys in some areas that target specific rare species. The distribution of species records is somewhat clustered, with largely unsurveyed areas interspersed with well surveyed areas (which tend to be in more accessible parts of this largely remote landscape; Fig. 2).

### Analysis

To model species’ distributions, we used boosted regression trees (BRTs, a machine learning technique) in R 3.4.0 (R Core Team 2018), with binary presence–absence data as the response. With such a complex dataset and largely unknown relationships (especially regarding geodiversity) over many different geographical contexts with variable collinearities and interactions, using a machine learning algorithm was preferable to a deductive modelling approach. Additionally, BRTs explicitly consider interactions between variables, which can point towards important combined effects, as well as dealing with non-linearity and collinearity reasonably well relative to other methods (Elith et al. 2008; Dormann et al. 2013). We produced models for the whole of the study area, and for the north, east, south, and west individually, to determine whether patterns were comparable in different areas of this landscape (Fig. 2). Results are presented for the whole area, unless they differed substantially between sections.

We used gbm.step (gbm 2.1.1 package in R) to implement the BRTs (Ridgeway 2017). This function uses shrinkage procedures as each tree is added, helping to control the number of terms, to produce a more parsimonious model. It tries to avoid overfitting by using regularisation methods. To study an individual predictor’s model effects, the contribution (relative model influence) of each predictor on the model outcome was obtained. These were scaled to add to 100, where a value of 100 for a predictor means that only that predictor contributed to the final model. To aid interpretability of the results, we calculated the correlation between a predictor and the response variable, and applied the direction of the relationship to the model contribution value, so that negative values represent negative relationships.

Most default parameters within gbm.step were maintained. The tree complexity of 3 allowed up to three-way interactions (Elith et al. 2008). The bag fraction was 0.5, and the preferred learning rate was 0.05, which was occasionally reduced to 0.01, 0.005 and 0.001, in sequence, according to data requirements. Predictors contributing < 10% (or sometimes < 7.5% where the former removed almost all variables) were removed from the initial model, which was rerun with the simplified predictor set to produce the final results.

As well as evaluation from internal fit statistics (self-statistics; SS), model performance was assessed using 10-fold cross-validation (CV) in the gbm package. CV randomly subsamples the data ten times according to the user-defined bag fraction (here 0.5, i.e. 50%) and tests the model on this held-back portion of data. The mean correlation between the training and each testing dataset is then reported and we additionally reported the area under ROC curve (AUC) values. For display purposes in the results, we multiplied SS, CV, and AUC values, which were originally between 0 and 1, by 100: a value of 100 would be a model that explains all of the variation in the data (SS) or predicts perfectly to a subset of data in the same area (CV), whilst 0 indicates a very poorly fitted or predicting model.

Analyses were run for multiple combinations of climate, topography, and geodiversity variables (Table 2) to assess the change (using Mann–Whitney U tests) in model performance (SS, CV, and AUC) when geodiversity data were added.

**Table 2:** Predictor sets used for Boosted Regression Tree Modelling

Model number	Variables used in model
1	Climate + Topography (i.e. traditional SDM variables)
2	Climate + Topography **+** **Hydrology + Landforms**
3a	Climate + Topography + Hydrology + Landforms** + Materials − source**
3b	Climate + Topography + Hydrology + Landforms **+ Materials − mineralogy**
4a	*Climate *+* Topography *+* Hydrology *+* Landforms *+***Landforms *×* Source***
4b	*Climate *+* Topography *+* Hydrology *+* Landforms *+***Landforms *×* Mineralogy***

Bold shows added or modified variables at each stage; italicised content = variables combined (x) together to create new data. Table 1 shows details of which variables are in each of the variable categories presented below

## Results

Many individual species distribution models demonstrated some statistical improvement upon the addition of various geodiversity data to a basic SDM (i.e. Model 1; climate and topography). Specifically, between Model 1 and Model 2 (i.e. addition of hydrology and landforms to basic SDM), increases were seen in self-statistics (internal fit) for 25% of rare and 78% of common species; in CV mean (predictive ability) for 68% of rare and 71% of common; and for CV AUC for 63% of rare and 71% of common SDMs (Table 3). Mann–Whitney U tests showed that for all common SDMs’ results together, there were statistically significant increases only between Model 1 and 2 for SS and CV, Models 1 and 4a for SS, CV, and AUC (addition of landforms and merged landform-mineralogy data), and Models 1 and 4b (addition of landforms and landform-mineralogy data). However, such significant improvements were not seen between Model 2 and 4a and 4b (i.e. adding combined landform data when the standard landform data were already included). Many individual SDMs in these classes and for rare species, however, showed statistical improvement even where a significant improvement across all models was elusive, but not to the extent seen in Table 3 after the addition of just landform and hydrology data between Models 1 and 2.

**Table 3:** A summary of model change between Model 1 (standard SDM) and Model 2 (addition of landforms and hydrology) for rare and common species; values show how many models were improved

	Number of paired models	Did SS improve between Model 1 and Model 2?			Did CV mean improve between Model 1 and 2?			Did CV AUC improve between Model 1 and 2?		
		Yes	No	% of models improved	Yes	No	% of models improved	Yes	No	% of models improved
Common	505	393	112	77.82	356	149	70.50	358	147	70.89
Rare	76	19	57	25.00	52	24	68.42	48	28	63.16

When considering all models together, self-statistics (SS), cross-validation statistics (CV), and CV AUC values showed a significant improvement for common SDMs

**Fig. 3:**
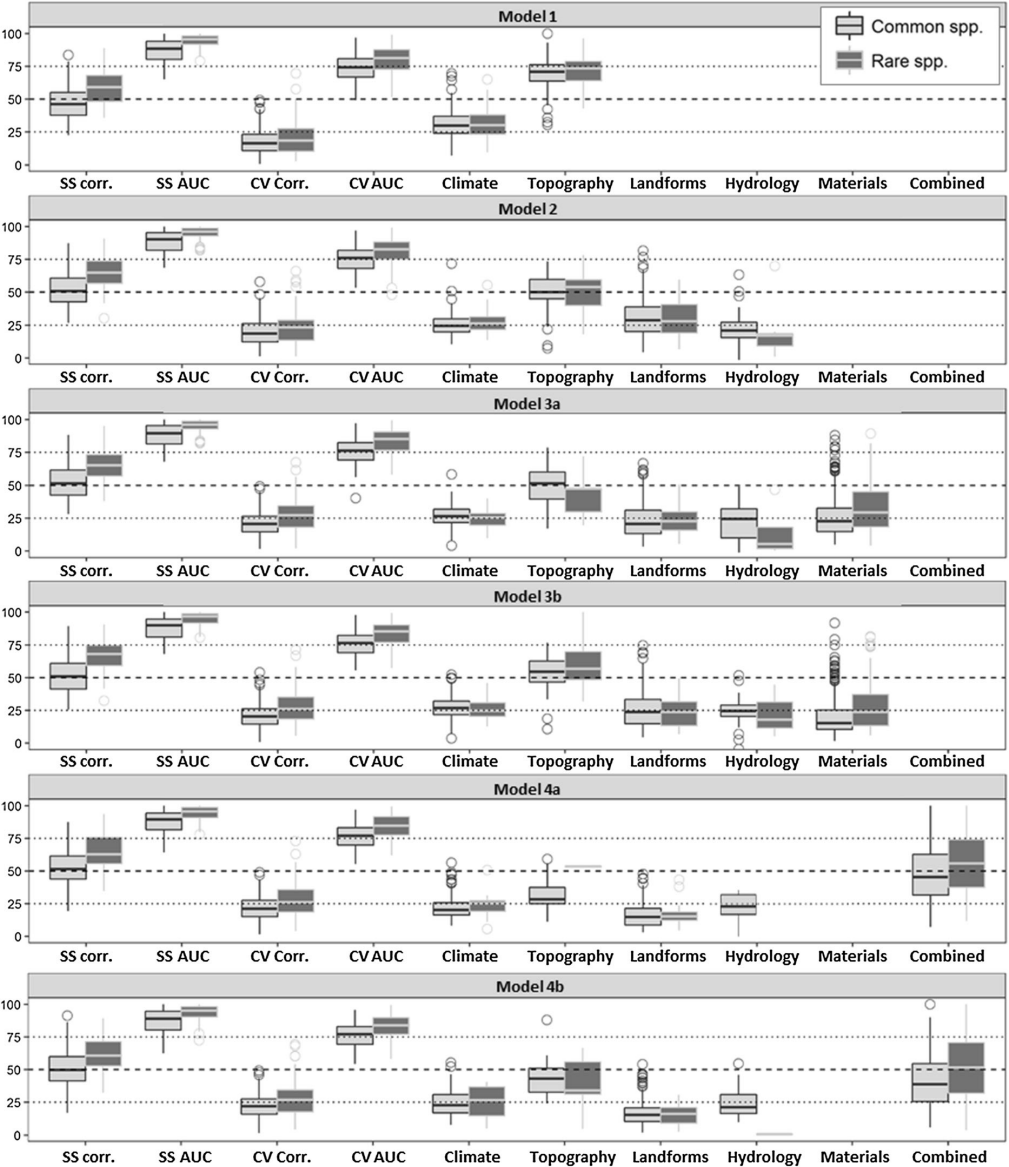
SDM statistics and absolute model contributions (y axis; 0–100) from each predictor set and sub-set (Table 2) for all common (light grey) and rare (dark grey) species across the whole of the Cairngorms National Park. Model statistics (SS, CV, AUC) have been multiplied by 100 for plotting but are normally between 0 and 1. Appendix S4 shows this same figure, but with model contributions modified to reflect negative relationships

In Models 4a and 4b (using the combined landform-material data), contributions to SDMs from all geodiversity components (GDCs) is clear (Fig. 3), even where significant model improvements were not observed. Topography data (mean and range in elevation and slope) typically dominated the SDMs. Contributions from climate (annual precipitation and annual mean temperature) and then landforms were comparable with one another. A clear decrease in model contribution was seen with topography upon the addition of landform and hydrology data (i.e. between Models 1 and 2; Fig. 3) and a much smaller decrease in model contribution from landforms upon the addition of the combined landform-material data. Climate was consistent in terms of its contribution to explaining variance, with a median of around 25% for rare and common species across all models.

Geodiversity variables frequently appeared amongst the dominant model predictors for Models 4a and 4b. Climatic and topographic variables generally defined the most frequent dominant model predictors (i.e. the predictors that explained the most variance), relating to species’ distributions in different directions, with mean temperature and mean elevation more often relating negatively to species; distributions and slope standard deviation, and a number of GDCs more commonly relating positively to species’ distributions. For example, as sedimentary alluvial and glacigenic spurs, and pits in Model 4a and rivers, clay-silica spurs and pits, basic slopes, and calcium carbonate slopes in Model 5a. Responses across each species were highly idiosyncratic, however. Climate and topography also defined the most common and strong model interactions with one another (e.g. mean temperature and annual precipitation with mean elevation), with some interactions also between topography and certain landforms (e.g. spurs and elevation; rivers and mean elevation; Table 4).

**Table 4:** The frequency of dominant model predictors with either positive or negative relationships with species’ distributions

	(a) Geodiversity: combined landform–source predictors (Model 4a)		(b) Geodiversity: combined landform–mineralogy predictors (Model 4b)	
	Common SDMs (n = 499)	Rare SDMs (n = 73)	Common SDMs (n = 500)	Rare SDMs (n = 75)
Most dominant POSITIVE model predictors				
1	Sed. alluvial spur (44, 19.37%)	Elev. (mean) (9, 41.14%)	Slope (SD) (41, 21.96%)	Elev. (mean) (16, 34.62%)
2	Temp. (mean) (43, 18.28%)	Slope (SD) (5, 32.41%)	Temp. (mean) (40, 18.52%)	CaCO_3_ slope (7, 27.3%)
3	Slope (SD) (35, 20.46%)	Met. Sed. Gen. hollow (5, 23.59%)	Pit (38, 23.17%)	Slope (SD) (5, 31.03%)
4	Pit (30, 18%)	Met. Sed. Gen. valley (4, 26.16%)	River (34, 17.53%)	CS pit (4, 30.37%)
5	Sed. Glaci-gen spur (27, 18.66%)	Sed. Glaci-fluv peak (4, 24.97%)	CS spur (32, 21.33%)	Basic slope (4, 27.68%)
Most dominant NEGATIVE model predictors				
1	Elev. (mean) (261, − 27.71%)	Precip. (mean) (14, − 20.04%)	Elev. (mean) (289, − 30.75%)	Elev. (mean) (13, − 27.89%)
2	Precip. (mean) (104, − 20.86%)	Temp. (mean) (12, − 16.77%)	Precip. (mean) (85, − 24.62%)	Precip. (mean) (13, − 19.16%)
3	Temp. (mean) (18, − 14.15%)	Elev. (mean) (8, − 29.2%)	Temp. (mean) (21, − 14.55%)	Temp. (mean) (11, − 17.94%)
4	Elev. (SD) (11, − 20.25%)	Slope (mean) (4, − 14.34%)	Elev. (SD) (20, − 23.93%)	Elev. (SD) (3, − 15.2%)
5	Slope (mean) (10, − 17.33%)	Met. Sed. Gen. slope (3, − 25.84%)	Slope (mean) (8, − 25.78%)	UB slope (2, − 22.88%)
Most frequent dominant interactions (selected)				
1	Precip. (mean) × Elev. (mean)	Precip. (mean) × Slope (SD)	Precip. (mean) × Elev. (mean)	Temp × Elev. (mean)
2	Slope (SD) × Elev. (mean)	Temp × Elev. (mean)	Slope (SD) × Elev. (mean)	Precip. (mean) × Slope (SD)
3	Temp × Elev. (mean)	Elev. (mean) × Met. Sed. Gen. Hollow	Temp × Elev. (mean)	River × Slope (mean)
4	Spur × Elev. (mean)	Hollow × Sed. Glaci-gen slope	Basic slope × Elev. (mean)	Slope (SD) × Elev. (mean)
5	River × Elev. (mean)	Met. Sed. Gen. valley × Elev. (mean)	Spur × Elev. (mean)	Slope (SD) × Slope (mean)

Structure: Predictor (count, average model contribution), where ‘count’ is the number of times that a predictor was the main predictor in the species’ distribution models (‘SDMs’). Selected dominant and most frequent interactions are also provided. A full table of results containing each species and the top five positive and negative predictors, as well as the model fit statistics and pivot tables, is provided in Appendices S5 and S6. If there was a joint ranking for position number 5 (i.e. two predictors were dominant in the same number of models), then the one with the greatest average contribution was used

*CaCO*_*3*_ calcium carbonate, *CS* clay/silica or silica/clay, *Elev.* elevation, *Gen.* generic, *Glaci*-*gen* glacigenic, *Glaci*-*fluv* glacifluvial, *Met* metamorphic, *Prec.* annual precipitation, *River* river length, *Sed.* sedimentary, *SD* standard deviation, *Temp* annual mean temperature, *UB* ultrabasic

Rather than attempt to detail the results of every SDM here, we present a few examples in relation to known ecology, from Models 4a and 4b (Table 5; the full set of species-by-species results is included in Appendices S5 and S6). Many SDMs produced results in-keeping with species’ known ecology. For example, for *Viola canina* (Heath Dog Violet), known to live mainly in heathlands (dry and wet) and prefer acidic substrates, we found a positive association with valley sides and alluvial fan materials and with clay-silica valley landforms. Meanwhile, *Saxifraga hypnoides* (Mossy Saxifrage), a species listed on the area’s Rare Plants Register, known to prefer rock ledges, shade and basic substrates was positively associated with hollow (specifically magnesium carbonate and clay-silica hollows) and slope landforms, and areas with steeper slopes and a greater standard deviation in slope. *Juncus trifidus* (Highland rush), which is known to be is associated with high, wind-swept plateaus, provides an example of an SDM driven almost entirely by topography and climate.

**Table 5:** Selected plants from wider SDM results (same species repeated in corresponding row for source [4a] and mineralogy [4b] analyses)

	Species	Rare/common (SS corr)	Dominant predictor (positive) (%)	Second predictor (positive) (%)	Dominant predictor (negative) (%)	Second predictor (negative) (%)	Main modelled interaction (a × b)	Notes on ecology (and status) of this species
Landform-source data (Model 4a)	*Viola canina*	Rare (0.63)	Sed. alluvial fan valley (11.65%)	Sed. mire or bog slope (7.85%)	Elev. (SD) (− 15.74%)	Slope (mean) (− 9.04%)	River × Sed. alluvial pit	Dry or wet heaths. Severe declines since 1950s
	*Spergularia rubra*	Common (0.59)	Met. Gen. hollow (19.73%)	Sed. glaciofluvial slope (10.26%)	Elev. (mean) (− 22.93%)	Precip. (mean) (− 11.64%)	Sed. glaciofluvial hollow × Sed. glaciofluvial slope	Free-draining sandy or gravelly ground
	*Saxifraga hypnoides*	Rare (0.61)	Met. Sed. Gen. hollow (42.79%)	Slope (mean) (28.35%)	NA	NA	Slope (mean) × Met. Sed. Gen. hollow	Damp rock ledges; boulders; screes
	*Carex atrata*	Rare (0.75)	Met. Sed. Gen. valley (26.62%)	Slope SD (25.47%)	NA	NA	Sed. weathering hollow × Slope (SD)	Ungrazed areas; faces of calcareous cliffs
	*Juncus trifidus*	Common (0.69)	Elev. (mean) (37.2%)	Elev. (SD) (8.64%)	Temp. (mean) (− 14.44%)	Igneous intrusive hollow (− 3.55%)	Ig. intrusive spur spur × Elev. (mean)	Wind-swept plateaus, lichen-rich crevices
Landform-mineralogy data (Model 4b)	*Viola canina*	Rare (0.69)	CS valley (9.31%)	Organic slope (7.08%)	Elev. (SD) (− 17.42%)	Elev. (mean) (− 10.29%)	Silica valley × Elev. (SD)	Acid habitats
	*Spergularia rubra*	Common (0.67)	CS slope (15.74%)	CS hollow (13.86%)	Elev. (mean) (− 26.15%)	CS pit (− 16.07%)	Precip (mean) × Elev. (mean)	Acidic sands and gravels
	*Saxifraga hypnoides*	Rare (0.57)	MgCO_3_ CS hollow (32.15%)	Slope (SD) (28.88%)	NA	NA	Lake area × Slope (SD)	Damp rock ledges; partial shade; base-rich substrates
	*Carex atrata*	Rare (0.77)	Hollow (29.96%)	Slope SD (25.75%)	River (− 9.35%)	NA	River × Slope (SD)	Ungrazed areas; faces of calcareous cliffs
	*Juncus trifidus*	Common (0.68)	Elev. (mean) (39.62%)	Slope SD (8.79%)	Temp. (mean) (− 13.56%)	Acid hollow (− 5.59%)	Elev. (SD) × Elev. (mean)	Wind-swept plateaus, lichen-rich crevices

*CS* clay/silica or silica/clay, *Gen* generic, *GF* glaciofluvial, *Ig.* igneous, *MgCO*_*3*_ magnesium carbonate, *Met* metamorphic, *River* river length, *SD* standard deviation, *Sed.* sedimentary, *Temp.* annual mean temperature

Rare or common according to the designation used in the ‘Rare Plants Register’ for the Cairngorms National Park, within which the definition of ‘rare’ is relatively broad (Amphlett 2012)

Notes on the ecology of each species principally from the ‘New Flora of the British Isles’ (Stace 2010) and/or www.brc.ac.uk

## Discussion

Our findings support the notion that geodiversity data (i.e. explicit landforms, surface materials, and hydrology) can improve traditional species distribution models that use only climate and basic topographic metrics; this research extends the limited body of existing research linking geodiversity to biodiversity. The greatest improvements, in terms of model evaluation statistics, were seen when hydrology (rivers and lakes) and automatically-generated landform data from geomorphometry were added to basic SDMs (topography and climate). Information on the coverage of surface materials in each grid cell (parent material and mineralogy) and the datasets combining landforms and materials resulted in no significant improvements across all models; many individual SDMs, however, showed an improvement, demonstrating idiosyncrasies between species.

The explanatory power of geodiversity, overall, was greater than previously found across most of Britain for species richness at similar landscape extents of around 25 km in diameter (Bailey et al. 2017). The existence of specific geodiversity contributions for explaining distributions of individual species (both ‘rare’ and ‘common’) represents a significant advancement in the context of the ‘Conserving Nature’s Stage’ (CNS) research agenda (Lawler et al. 2015). Indeed, in linking individual species’ distributions to specific geofeatures, we move correlative SDMs closer to the real ecologies of species.

Improved knowledge of species’ relationships with specific geofeatures across spatial scales is likely to be important in the context of climate change, whether it be due to indirect (e.g. direct effects on physical soil properties and physical processes such as snow melt) or direct (e.g. species’ thermal tolerances) climate impacts (Shaw and Thompson 2006; Brazier et al. 2012). For the many SDMs that were dominated by climate, geofeatures still showed additive effects. Meanwhile, those species for which geofeatures were most important may be safeguarded against climatic changes where these features are proactively managed and considered in protected area planning. The presence of specific materials (geology and soil), landforms and hydrological systems may help to ensure continuation of particular species; geodiversity has been linked to species’ persistence and adaptation to climate change in North America, for example (Anderson et al. 2014; Albano 2015; Magness et al. 2018). The importance of topography-driven microclimatic heterogeneity for buffering species against climate change has recently been demonstrated (Suggitt et al. 2018), which, along with the present study’s use of elevation and slope, supports the continued value of such topographic metrics.

For those species whose distributions were dominated by climate, but where geofeatures were still contributory, indirect climatic effects may be manifested within these features and their importance may not be proportional to their modelled contribution, given known indirect effects through soils and landforms. For example, increases in monthly minimum temperatures are likely to affect species not only directly, but also via changes to geomorphological and soil processes, such as changes in weathering, erosion, disturbance events (Viles et al. 2008; Virtanen et al. 2010), snow melt (Kankaanpää et al. 2018) and water storage and transfer (Brazier et al. 2012), and changes in microbial communities (Zogg et al. 1997). Many such processes are implicitly incorporated in our models through the explicit inclusion of geodiversity, which may act as a proxy for microclimate and fine-scale resource availability (Hjort et al. 2012; Anderson et al. 2014; Tukiainen et al. 2017b). In the context of future climate change, these findings may therefore be significant in understanding species’ distribution changes, given the expected temperature and rainfall increases in this part of the United Kingdom (Werritty 2002). However, such processes have not been explicitly modelled here, and more work is needed in this area.

Geofeatures relating to pit landform coverage (i.e. areas of depressed land surrounded by relatively flat ground) were frequently a dominant predictor that had a positive relationship with species’ distributions. This variable is likely to be hydrologically relevant and may represent unmapped lochans (small lakes), ponds, bogs and kettleholes, for example. These would generally be very moist and may form temporary ponds, which have been linked closely to species’ distributions (Vandvik et al. 2005; Hjort et al. 2015). They are, however, likely to be important beyond their hydrological properties because indented surfaces can provide protection from high winds, humans and grazing animals (Vandvik et al. 2005).

Similar considerations apply to mountain hollows, which provide shade and shelter, as well as rocky outcrops. Hollows (either in themselves or in combination with land surface materials) frequently contributed to our SDMs. Late-lying snow patches in the Cairngorms are common, providing important habitats (Gimingham 2002), and hollows affect spatiotemporal snowmelt and moisture patterns, which has been noted in higher-latitude landscapes (Litaor et al. 2008; Kankaanpää et al. 2018). Quantifying landform morphology and combining these data with information on land surface materials may provide more useful information for managers studying species’ distributions compared to using crude DEM-derived metrics such as slope and the topographic wetness index (TWI), for example. TWI, for example, may fail to represent hydrology and soil moisture levels effectively because of local edaphic and geological conditions (Kopecký and Čížková 2010).

Our findings relate to the body of research on ‘CNS’, in which the focus of conservation is on the areas capable of supporting higher biodiversity because of inherently higher geodiversity (Anderson and Ferree 2010; Lawler et al. 2015). Geodiverse locations are thought to improve species’ ability to adapt and persist in the face of climatic changes, which is supported by studies of microclimatic refugia and buffering (Lenoir et al. 2013; Lawler et al. 2015; Suggitt et al. 2018). Indeed, considering explicit geofeatures’ edaphic, hydrological, and solar properties (rather than just using general topographic metrics) in the context of buffering and microclimates provides a clear next step to help with the targeted management of these geofeatures for biodiversity conservation. Geodiverse areas, however, are not always well represented by protected area networks (Albano 2015; Ordonez et al. 2016).

An overall geodiversity metric may identify an area as a good ‘stage’ upon which to conserve biodiversity generally (i.e. where the identities of the species present are less important than overall biodiversity), but give little information as to why that site is good, beyond that it is simply ‘geodiverse’: hydrological features, a certain soil type, or specific landform might be driving the richness-geodiversity relationship. Therefore, targeting specific features is likely to be of value to management efforts. If so, explicit links have to be made between specific geofeatures and species across multiple taxa and scales and geodiversity metrics should, where possible, be accompanied with geofeature-specific analyses, such as in the present study. More frequently considering individual species’ distributions would be beneficial for empirical work, as well as for practical conservation. As part of conservation efforts, such empirical studies using geodiversity should support proactive, rather than reactive, planning and management, including accounting for connectivity, through incorporation of geodiversity (Magness et al. 2018).

Reserve management also needs to account for biological interactions. It is not possible without fine-scale studies to ascertain the extent to which particular landforms (e.g. depressions such as pits and hollows) have contributed to our models because of the biological protection they offer, but, for example, we saw model contributions from hollow and valley predictors for *Carex atrata’s* distribution model, which is known to favour ungrazed areas. Grazing is particularly relevant to management in Scotland, and for British uplands more widely (especially in terms of deer and hares). Previous work suggests that some landforms can indeed protect plants and lichens from grazing (Gulliver 2013; Moore and Crawley 2014), and some of our results may reflect this. Deer numbers in Scotland are thought to be stabilising after sharp increases since the 1960s, while numbers are actively being managed and reduced in some parts of the Scottish Highlands (Edwards and Kenyon 2013). Fenced-off areas could reveal much about the ability of landforms to shelter species from herbivores. For example: how do geofeature–biodiversity and geofeature–species relationships differ between grazed and ungrazed areas?

### Data considerations

Data produced automatically using geomorphometry were of great value in this study and required a relatively fine-scale DEM and open source GIS software (GRASS Development Team 2018). Such techniques will no doubt become more common as algorithms develop and the user base of open source GIS software expands. We also combined these morphology data with 1:50,000 scale geology maps to produce a semi-automated dataset that was more conceptually sophisticated than using morphometry data alone (though, this extra data effort did not translate into quantitative model improvements). However, this is still very different to using professionally-mapped geomorphology data where knowing about the presence of a specific feature (e.g. an esker or kame terrace) can immediately provide the modeller with information on fine-scale abiotic processes and conditions (Hjort and Luoto 2010). It therefore remains an open question as to whether automatically mapped geomorphometry data are more or less useful than cheaper and easily-obtainable geomorphometrics for understanding species’ distributions and biodiversity patterns.

In the absence of such professional geomorphology maps, the techniques used in our study may provide an intermediate solution and additional knowledge: automated geomorphometry combined with existing geological maps. We therefore suggest that information on surface materials should be used alongside morphology data where possible, despite the limited model improvements seen, because they added conceptual value to models for certain species, even though improvements to the models were not seen across all SDMs. These surface material data may be hard to extract automatically where they do not exist, but remote sensing techniques have much potential in this context (see Discussion in Bailey et al. 2017) and national and global databases are growing (e.g. Hengl et al. 2017).

## Conclusions and Future Directions

Our study represents a clear progression in the use of spatially explicit geodiversity data within the broader body of environmental heterogeneity research at the landscape scale. We saw consistent model improvements after incorporating morphological landform data into SDMs and recommend wider consideration of such data. These data were straightforward to produce and added much conceptual value around understanding why species were found in certain places. Combining these data with surface material properties relating to source and mineralogy added further conceptual value, but quantitative model improvements were less consistent. A greater awareness of geofeatures in conservation and management will be beneficial in the face of environmental change, to enable more informed decisions about protected area planning and management. Geodiversity as a concept provides a tangible means to achieve this and will allow for the targeting of explicit, identifiable features on the ground that we can relate to abiotic properties for biodiversity conservation.

We found that predictive ability (measured using cross-validation) was generally low, which may be due to the quantity of species observations and shortage of training data or, alternatively, a real effect of species’ dispersal limitations in this landscape (Guisan and Thuiller 2005; Zurell et al. 2009). However, internal fit (self-statistics) values were generally high. It therefore remains an open question as to whether these geodiversity data can improve models’ predictive ability, or whether they are most suited to improving models in a given place and time.

It would be useful to improve our understanding of the abiotic properties surrounding specific geofeatures (e.g. are some geofeatures’ properties especially relevant in the context of microclimate buffering, sheltering, and resource provision?); develop a fuller understanding of different types of geodiversity data and which might be useful in different contexts (e.g. are professionally mapped geomorphology data significantly better at predicting species’ distributions than geomorphometry data?); and assess the role of geodiversity at a greater range of spatio-temporal scales and for multiple taxa (e.g. are geodiversity data more relevant for species with specific life history traits?). Essentially, further integrating geodiversity data into science and policy, as well as identifying when and where (geographically and taxonomically) different components of geodiversity are of the greatest value should be key considerations moving forwards.

## Electronic supplementary material

Below is the link to the electronic supplementary material.

Supplementary material 1 (PDF 1121 kb)

Supplementary material 2 (XLSX 577 kb)
