# Supplementary material for: Models of upland species’ distributions are improved by accounting for geodiversity
Source: Landsc Ecol. 2018 Oct 28;33(12):2071–87. doi: 10.1007/s10980-018-0723-z (PMC6404796; doi:10.1007/s10980-018-0723-z)

SUPPORTING INFORMATION

for

Joseph J. Bailey*[1], Doreen Boyd[1], and Richard Field[1]:

"*Models of upland species' distributions are improved by accounting for geodiversity*"

* Email: josephjbailey@outlook.com

[1] School of Geography, University of Nottingham, University Park, Nottingham, NG7 2RD, United Kingdom

CONTENTS

Appendix S1: Further information on the Cairngorms National Park 2
References for Appendix S1 3
Appendix S2: Maps of geodiversity components (GDCs) 4
Appendix S3: Land surface material variables summary 6
Appendix S4: Same as Fig. 3, but showing negative values 7

Appendix S1: Further information on the Cairngorms National Park

(There is some overlap with the main text)

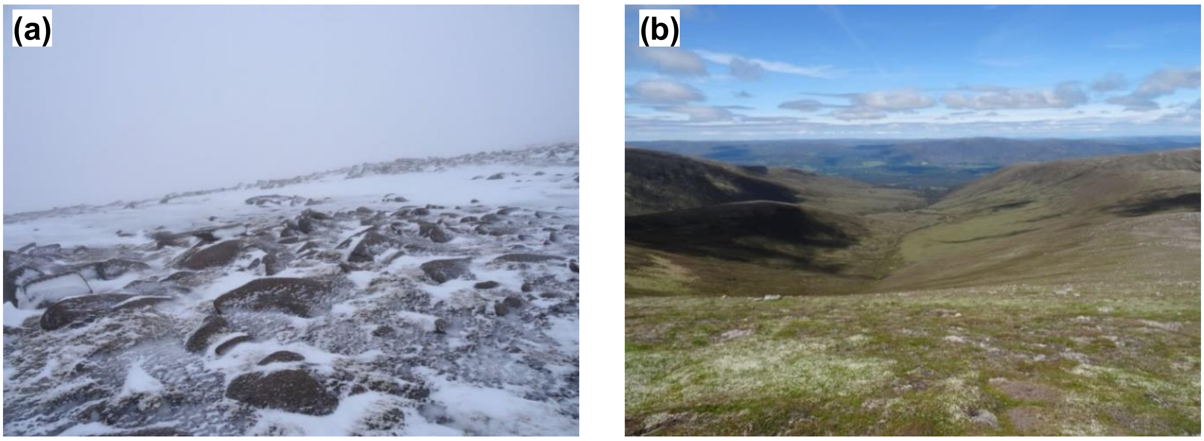

Photographs were taken in two different locations within the Cairngorms National Park at two times of the year, showing the diverse annual climatic conditions that the flora and fauna face. **(a)** Nearing the summit of the mountain Cairn Gorm (photograph taken at altitude of ~ 1, 150 m) in winter, the mountain after which the national part is named (January, 2016). **(b)** Looking approximately north-west and standing downslope of Sgòran Dubh Beag in the Invereshie and Inshriach National Nature Reserve (June, 2013). © Joseph J. Bailey.

The Cairngorms National Park in the Central Highlands of Scotland. It is the highest area in Great Britain and is broadly illustrative of British upland landscapes. Additionally, the Cairngorms contains four of the five largest mountains in the UK and there are 52 summits over 900m. Britain's upland landscapes are often inherently geodiverse areas, with intricate geological and hydrological features and complex topographies. However, they have been impacted by humans since deforestation in Neolithic times, with more recent activities including tourism and intensive management for agriculture, grouse hunting and deer stalking: these activities can be beneficial but excessive burning of heath and grass can degrade affected areas (Burt et al., 2002). Meanwhile, grazing is a widely-reported issue in relation to hampering the growth of native flora (JNCC, 2002; Medina-Roldán et al., 2012; Moore and Crawley, 2014; Ross et al., 2016), as is the sensitivity of peat, which is important in the context of carbon storage and climate change (Worrall et al., 2003; Evans and Warburton, 2010).

The Cairngorms was largely formed during the Caledonian Orogeny from the late-Silurian to the early-Devonian (~ 425 Ma) and the area is formed of a granite pluton created by at least two intrusions, which contains mostly quartz and feldspar (Gordon and Sutherland, 1993; Hall et al., 2013). The present geomorphological assemblages are typical of a once-glaciated mountain area, with glacial erosion generally confined to the valleys and corries, dissecting the uplands (Hall et al., 2013). There are also many pre-glacial and non-glacial landforms, as well as those resulting from glacial erosion or deposition. The first glaciation of the Cairngorms was approximately 2.6 Ma, after which alternating glacial and periglacial conditions ensued (Clapperton, 1997; Hall et al., 2013). Pre-glacial landforms are extensive and include paleosurfaces and breaks of slope, topographic basins, valleys (the main valleys

predated the Pleistocene), domes, and tors (detailed by Hall et al., 2013). Glacial landforms are marked by sudden breaks from the gentler pre-glacial mountain forms and include corries and roches moutonnées, as detailed elsewhere (Sugden, 1968; Gordon and Sutherland, 1993), alongside periglacial features such as rock glaciers and boulder fields.

Deer are common and form an important part of the area's heritage. However, high numbers prevent native woodland regeneration and can negatively impact fragile habitats such as moss and lichen heaths and blanket bogs (Edwards & Kenyon, 2013). The 'rewilding' of various fauna, which are and will impact the existing species, presents an ongoing discussion in the Cairngorms, and the UK's uplands more generally (e.g. see Sandom et al., 2013).

In terms of human presence, about 16,000 people currently live in the Cairngorms across several lower-altitude settlements and isolated properties. Meanwhile, tourism accounts for 80% of the economy and there are thought to be around 1.4 million visitors to the area every year (Visit Cairngorms, 2016).

References for Appendix S1

Burt, T. P. et al. (2002). The British Uplands: Dynamics of Change. JNCC Report No. 319.

Clapperton, C.M., 1997. Greenland Ice Cores and North Atlantic Sediments: Implications for the Last Glaciation in Scotland. In: J.E. Gordon (Editor), Reflections on the Ice Age in Scotland. Scottish Natural Heritage, Edinburgh, pp. 45-58.

Evans, M., and Warburton, J. (2010). Geomorphology of Upland Peat: Erosion, Form and Landscape Change. Royal Geographical Society with IBG book series. Blackwell Publishing Ltd, UK

Gordon, J.E. and Sutherland, D.G. (1993). Quaternary of Scotland, Geological Conservation Review Series, No. 6, Chapman and Hall, London, pp. 695.

Hall, A. M. et al. (2013). Scottish Landform Examples: The Cairngorms – A Pre-glacial Upland Granite Landscape. *Scottish Geographical Journal*, **129**, 2–14.

Medina-Roldán, E. et al. (2012). Grazing exclusion affects soil and plant communities but has no impact on soil carbon storage in an upland grassland. *Agriculture, Ecosystems and Environment*, **149**, 118–123.

Moore, O. & Crawley, M. J. (2014). The natural exclusion of red deer from large boulder grazing refugia and the consequences for saxicolous bryophyte and lichen ecology. *Biodiversity Conservation*, **23**, 2305–2319.

Ross, L. C. et al. (2016). Sheep grazing in the North Atlantic region: A long-term perspective on environmental sustainability. *Ambio*, **45**, 551–566.

Sandom, C. J. et al. (2013). Rewilding the Scottish Highlands: Do Wild Boar, Sus scrofa, Use a Suitable Foraging Strategy to be Effective Ecosystem Engineers? *Restoration Ecology*, **21**, 336–343.

Sugden, D. E. (1968). The Selectivity of Glacial Erosion in the Cairngorm Mountains, Scotland. *Transactions of the Institute of British Geographers*, 45, 79–92.

Visit Cairngorms (2016). Cairngorms National Park Key Facts (online). Available at: http://visitcairngorms.com/keyfacts (last accessed September 2016)

Worrall, F. et al. (2003). Carbon budget for a British upland peat catchment. *The Science of the Total Environment*, **312**, 133–146.

Appendix S2: Maps of geodiversity components (GDCs)

Appendix S2a Hydrology

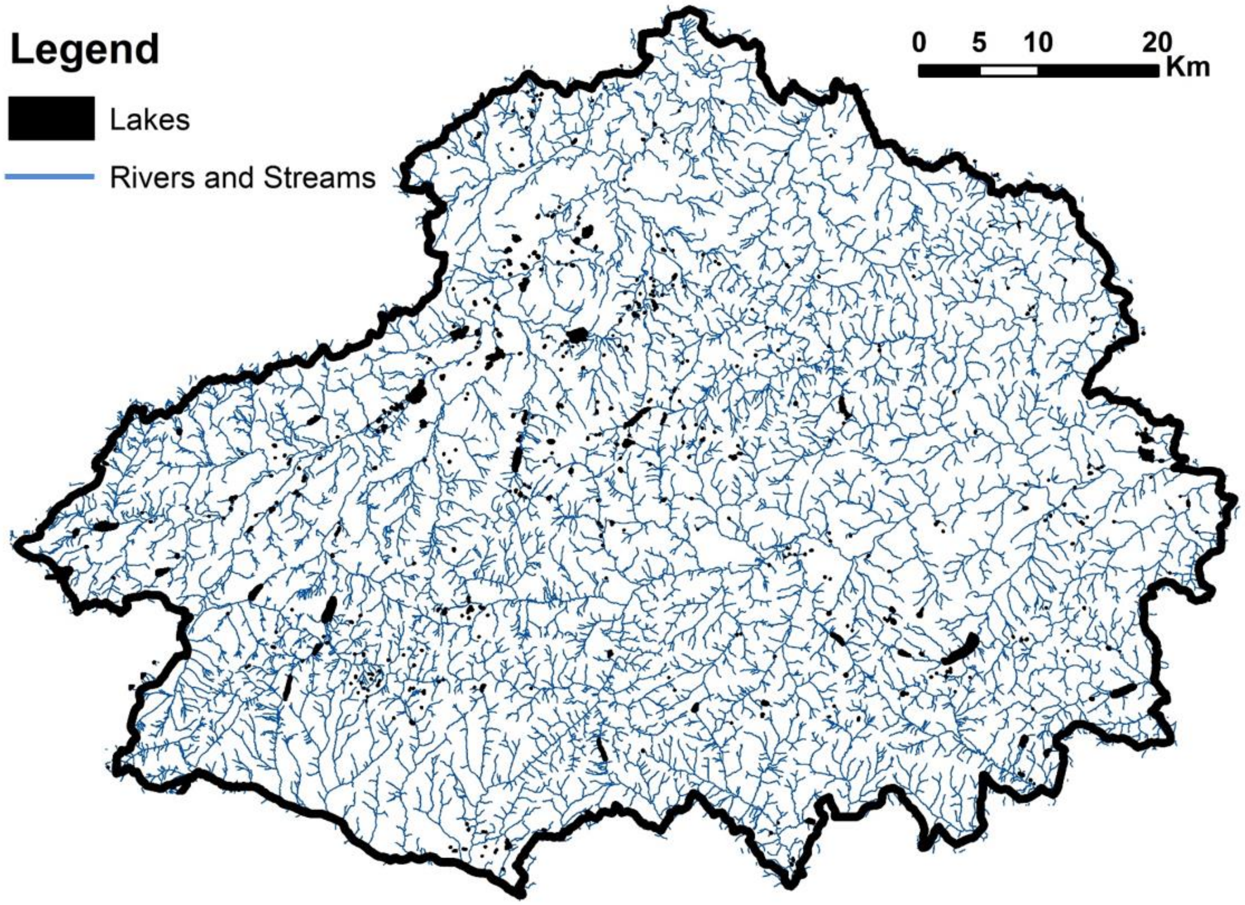

Appendix S2b Aggregated data examples (i.e. geomorphometry and hydrology data once summarised per 1km^2 grid cell).

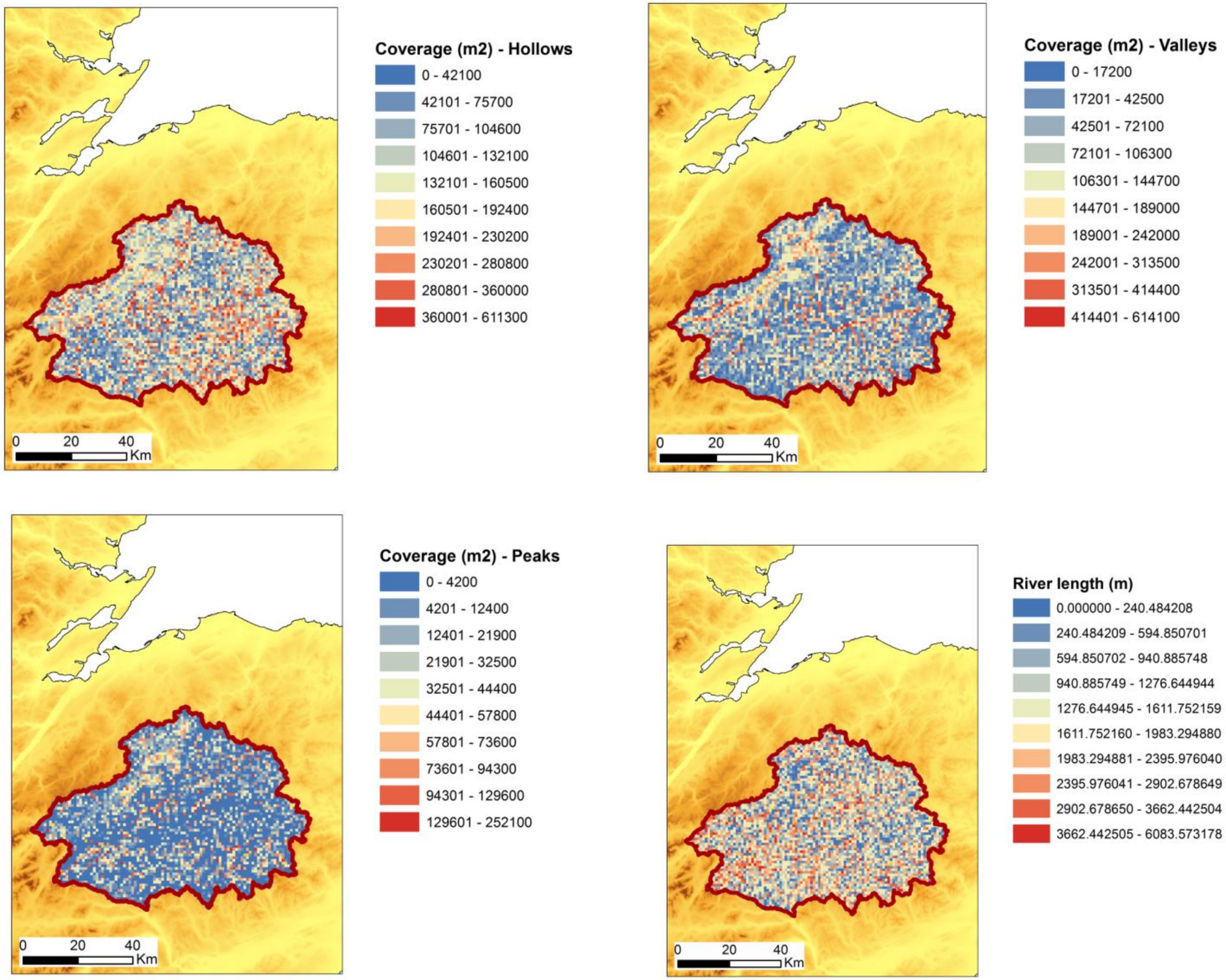

Appendix S3: Land surface material variables summary

Below are listed all of the materials that went into either the combined landform-source or landform-mineralogy datasets. Where these surface material data overlapped with a landform (peak, ridge, shoulder, spur, slope, footslope, hollow, valley, flat area, or pit), a new combined variable was created and its areal coverage used in the modelling (see Methods). Data source: British Geological Survey (see Table 1 in Methods). SED = sedimentary; IGN = igneous; MET = metamorphic.

SOURCE (genesis and rock type)
SED_ALLUVIAL
IGN_INTRUSIVE
MET_SED_GENERIC
SED_MIRE_OR_BOG
SED_ALLUVIAL(TERRACE)
SED_GLACIOLACUSTRINE
SED_GLACIOFLUVIAL
MET_IGN_INTRUSIVE
MET_GENERIC
MULTIPLE
IGN_EXTRUSIVE
SED_GLACIGENIC
SED_PERIGLACIAL
SED_GENERIC
IGN_MINERALISATION
SED_LACUSTRINE
SED_ALLUVIAL(FAN)
SED_FLUVIAL
MET_IGN_EXTRUSIVE
MET_IGN_VOLCANOCLASTIC
SED_LITTORAL_MARINE
SED_AEOLIAN
SED_MARINE
SED_WEATHERING
SED_LAGOONAL_MARINE
SED_RESIDUAL
IGN_VOLCANOCLASTIC
SED_PRECIPITATION

MINERALOGY
CLAY-SILICA
BASIC
SILICA-CLAY
ORGANIC
INTERMEDIATE
SILICA
ULTRABASIC
ACID
CLAY-SILICA-CACARBONATE
CACARBONATE
CACARBONATE-SILICA
CACARBONATE-CLAY-SILICA
SILICA-CACARBONATE
MGCARBONATE
CLAY-CACARBONATE
MGCARBONATE-SILICA-CLAY
CACARBONATE-MGCARBONATE

Appendix S4: Same as Fig. 3, but showing negative values.

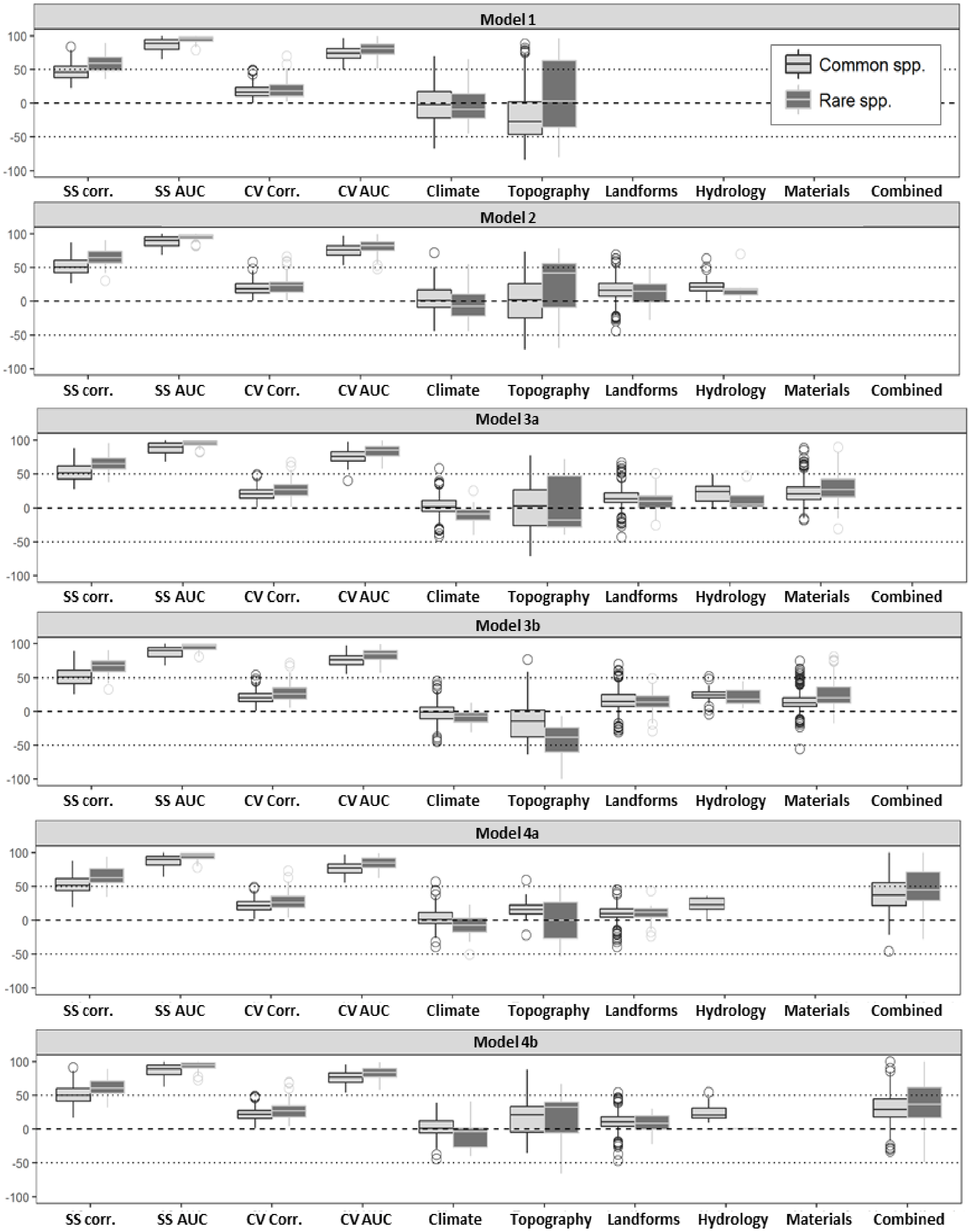

Supplement: Supplementary file 1 — Supplementary material 1 (PDF 1121 kb) [file 10980_2018_723_MOESM1_ESM.pdf]
